# Can money buy control of Congress?

**DOI:** 10.1371/journal.pone.0305846

**Published:** 2024-06-26

**Authors:** William Minozzi, Gabriel J. Madson, David A. Siegel

**Affiliations:** 1 Department of Political Science, The Ohio State University, Columbus, OH, United States of America; 2 RTI International, Research Triangle Park, NC, United States of America; 3 Duke University, Durham, NC, United States of America; Thammasat University Institute of East Asian Studies, THAILAND

## Abstract

Can a political party spend enough across electoral campaigns to garner a majority within the U.S. Congress? Prior research on campaign spending minimizes the importance of campaign heterogeneity and fails to aggregate effects across campaigns, rendering it unable to address this question. Instead, we tackle the question with a system-level analysis of campaign expenditures. First, using a flexible machine learning approach, we show that spending has substantial and nonlinear marginal effects on outcomes at the level of the campaign. Second, by aggregating these effects to the entire U.S. Congress, we show that large seat swings that change congressional control have, in the past, been possible for expenditure levels consonant with those presently observed after having removed the most extreme levels. However, this possibility appears to have faded over the past decade. Our approach also allows us to illustrate the often significant effects that eliminating campaign spending could have.

## Introduction

An overwhelming majority of Americans agree that money has more influence on politics than ever [1]. Money pays for advertising and get-out-the-vote operations, with media outlets regularly reporting that donors spend substantial sums on campaigns. Donors give to candidates in a largely ideologically-motivated manner and can strategically contribute to elections that may be more influential [2–4]. Such motivations propel insurgent candidates, like Bernie Sanders and Donald Trump, who run by opposing a system purportedly corrupted by money.

These concerns presume that campaign spending affects elections, but the science on that question is nuanced. One popular account of the scientific consensus on the question goes so far as to answer “Does campaign spending work?” with “Maybe. Sometimes. We’re not sure” [5]. On one side of the issue, some scholars argue that spending effects are small [6, 7], a position summarized in the claim offered in a recent review that it “has at best negligible impacts on election outcomes” [8]. On the other side of the issue, in a different recent review, Jacobson [9] argues that scholarship “leaves no room to doubt that campaign spending matters.” Moreover, down-ballot elections may behave differently from presidential ones, with the former showing a larger effect of television advertising [10] and employing more targeted online advertising [11].

Two common analytical choices contribute to a nuanced view of the effect of campaign spending. First, most research focuses on the seat-level, comparing effects for challengers and incumbents [6, 12, 13]. Such research does not address system-level effects, most notably control of Congress. Spending effects on individual seats might be small, yet still accumulate to large global outcomes. Second, the most plausible estimates of the causal effects of spending use field experiments on small numbers of races [6] or instrumental variables or panel data to identify local average treatment effects [14]. In addition to focusing on small, non-random subsamples, these approaches use linear models. In principle, local effects can be linearly extrapolated to yield back-of-the-envelope global estimates. However, because elections are contests [15], effects are largest in close races [13, 14], and this linear assumption can lead to incorrect system-level estimates.

We address both issues, in two steps. First, we model election outcomes, using kernel regularized least squares (KRLS) [16] to estimate seat-level marginal effects of expenditures, adjusting for a large number of covariates. By “effects” we do not mean to imply identification, but to match common usage of the term. “Effects” should be taken to mean expected differences in electoral outcomes between observed, rather than counterfactual, spending levels. KRLS is well-suited to modeling the relationship between spending and outcomes because it is flexible enough to incorporate nonlinearities and complex interactions, while avoiding overfitting. We show that spending has heterogeneous—and sometimes large—effects obscured by linear models, but consistent with the theoretical expectation of an inverted U-shaped curve, reaching its maximum when candidates spend equally [13, 17]. This similarity between theory and empirical estimates lends credence to the model.

Second, we use our models to simulate system-level outcomes—the number of seats held by each party—under hypothetical spending profiles. Specifically, we estimate seat counts for a large number of “leveling strategies” [18], in which either party is assumed to top-up their candidates’ spending advantage to some minimum level. We examine hypotheticals in which that minimum spending advantage ranges from the observed level to the 95^th^ percentile. The simulated seat shifts reach up to *one-third* of each chamber, easily enough to switch party control. Next, to examine the consequences of eliminating campaign spending, we estimate system-wide outcomes assuming all campaign expenditures are at minimal observed levels. We therefore operationalize our analysis of the possibility that money can buy control of Congress in terms of the existence of a set of leveling strategies that counterfactually could swing majority status in each chamber. Our simulated seat shifts indicate that present expenditure levels distort outcomes from a baseline comprising minimal spending. Democrats appear to benefit more from eliminating money from House elections, while Republicans benefit more in the Senate. Our results thus support popular perceptions about campaign spending. In Appendices E–H (S1 File), we document that our findings are robust to a large number of alternative research designs, including the use of different machine learning methods and amended sets of covariates.

Finally, we observe that the relationship between system-level outcomes and campaign spending has declined over the past four decades, and so increasingly large spending advantages would be necessary to affect control of Congress. We identify possible explanations for this observation, based on the evidence and models. One, at the seat-level, the observed gap between spending by the leading and trailing candidates has increased over time. Consequently, fewer seats fall at the top of inverted U-shaped curve, where spending effects are largest, and more fall in the tails, where effects are minimal. Two, seat-level total spending by both leading and trailing candidates has more than doubled over the past forty years. This fact matters because there are likely diminishing marginal effects to spending [9], for example from get-out-the-vote efforts and name recognition campaigns. Therefore, additional spending in more recent elections will have smaller—even negligible—effects than similar spending in earlier cycles.

## Campaign spending & electoral outcomes

Our analysis focuses on general elections in the U.S. House and Senate from 1980–2018. Our outcome variable is *Democratic Vote Share*, the proportion of major-party votes the Democratic candidate received. For dataset construction, we relied on campaign finance information from the Federal Election Commission (FEC), election data from the Clerk of the House, and contest covariates from Bonica’s Database on Ideology, Money in Politics, and Elections (DIME) and Jacobson’s challenger quality dataset. Specific discussion of the construction of our dataset can be found in Appendix B (S1 File). Our key independent variable is *Democratic Expenditure Advantage*, the difference between spending in support of the Democratic candidate and spending opposing them, including outside spending, in real 2016 dollars. We code outside spending as supporting/opposing a candidate if it supports/opposes the candidate or opposes/supports the candidate’s opponent. Positive/negative values reflect Democratic/Republican advantages. As discussed in Appendix C (S1 File), we use interaction terms to focus on the *Democratic Expenditure Advantage* for the middle 90% of its distribution, about ±$1.7 million in the House and ±$11 million in the Senate, but results are similar when we exclude the interactions or drop tail cases entirely. We do that as the observations in the tails may be misleading: there are likely decreasing returns to scale from campaign spending; values in the tails may emerge from wealthy, yet poor quality, candidates who go on to lose dramatically; and our hypotheticals of interest are concerned with the more conservative ranges indicated by the middle 90% of the distribution.

Expenditures and outcomes depend on candidate ideology, so we adjust for candidates’ common-space campaign finance (CF) scores [3] and the absolute difference between scores. Both spending and outcome depend on constituent preferences, so we adjust for *Democratic Presidential Vote Share* centered around the national vote. To account for decreasing returns to spending, we adjust for log *Total Expenditures* by major party candidates, super political action committees (PACs), and outside interests. We use dummy variables for election cycles to account for national tides [19], and dummies for party incumbency and open seats. Our House model includes *Challenger Quality* [12]. Our Senate model includes log *Voting Eligible Population* [20]. KRLS flexibly accounts for interactions between covariates—e.g., between candidate ideology and constituent preferences—when predictive, a fact we made use of when capturing decreasing returns via total spending. See Appendix C (S1 File) for model specifications and Appendix D (S1 File) for descriptive statistics.

### Effects of campaign expenditures on an individual campaign

**Fig 1:**
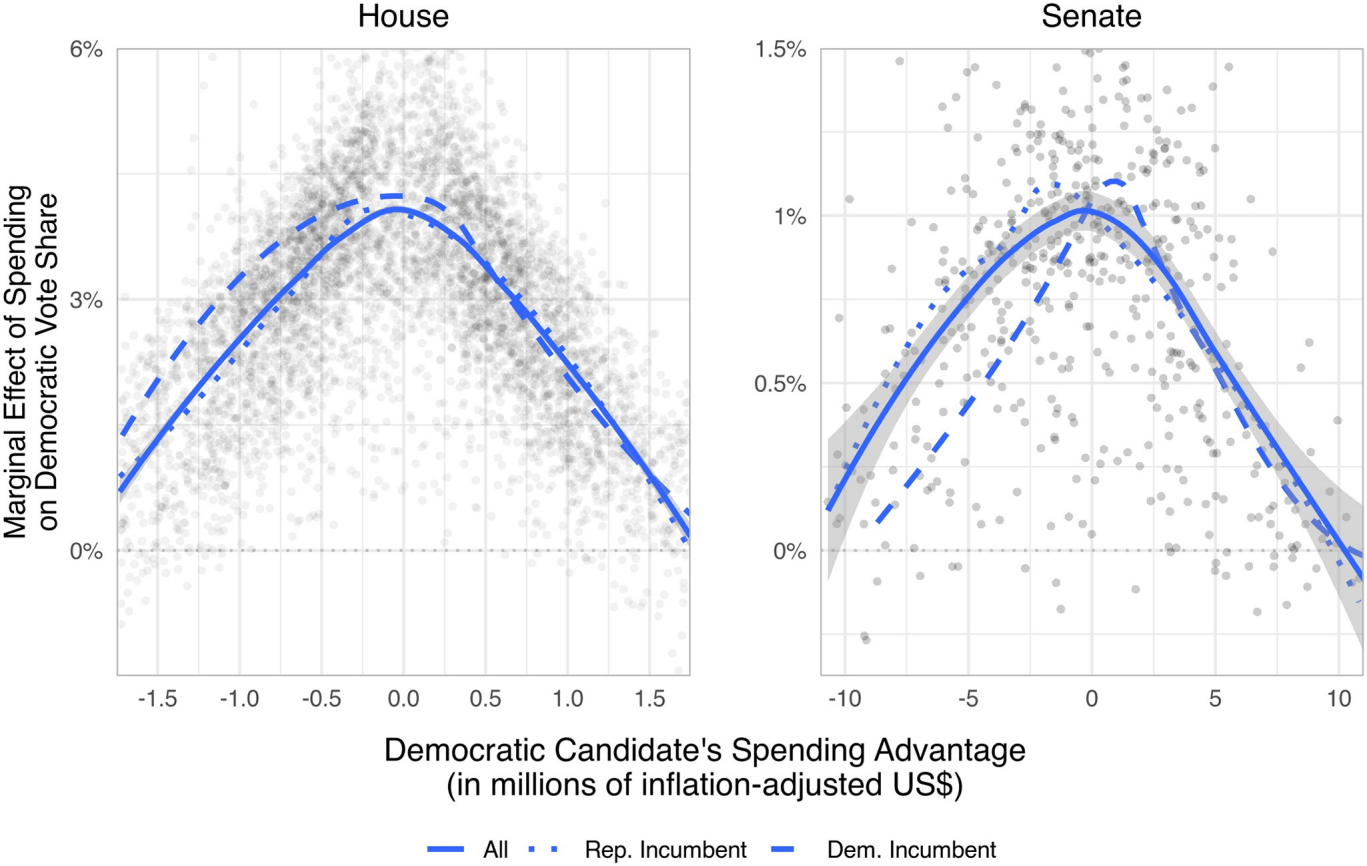
Money is most effective with spending near parity. Points are estimated marginal effects of *Democratic Spending Advantage* based on KRLS models. Lines are LOESS (locally estimated scatterplot smoothing) fits.

Campaign expenditure advantages appear to have substantial effects on an individual candidate’s election. Fig 1 plots seat-level marginal effects of *Democratic Spending Advantage* against the variable itself. Consistent with expectations from contests, there are inverted U-shaped curves for both chambers. In the House, the marginal effect approaches 4.5% near parity. In the Senate, it approaches 0.75%. All reported results are robust to alternative machine learning methods (Appendix E, S1 File), adjustment for lagged outcomes and spending (Appendix F, S1 File), and changing the outcome variable to a dichotomous measure of Democratic victory (Appendix G, S1 File). Note that the marginal effects of spending for incumbents are similar to those for challengers.

### Cumulative effects of expenditures

To estimate the cumulative effects of spending, we measure the areas under the curves in Fig 1. In the House, numerically integrating from the 5^th^ percentile (−$1.7M) to the 95^th^ percentile (+$1.7M) produces a 9% jump in *Democratic Vote Share*. In the Senate, similar integration, from −$11M to +$11M, yields a 10% jump. These increases suggest the potential global impact of spending. In 2018, about 24% of House seats were decided by margins less than 9%, and 34% of Senate seats by margins less than 10%.

Next, we simulate system-level effects. First, we estimate seat counts for a large number of “leveling strategies” [18], in which either party is assumed to top-up their candidates’ spending advantage to some minimum level. We examine hypotheticals in which that minimum spending advantage ranges from the 5^th^ percentile to the 95^th^ percentile, which covers the range from large Republican spending advantages to large Democratic spending advantages. Further details can be found in Appendix A (S1 File). For clarity, we illustrate our hypotheticals in two ways. To explore whether control of Congress could potentially be bought, we begin by showing the simulations for our most extreme hypotheticals within the central 90% of the distribution: one in which *Democratic Spending Advantage* is set to the 95^th^ percentile in each race, and another to the 5^th^ percentile. These amounts are large, but well within the range of present spending amounts. In real (2016) terms, these spending advantages would require average additional spending of about $450M per cyclein the Houseand $275M to $300M per cyclein the Senate. For comparison, in 2016 Republican candidates and outside groups collectively spent about $640M on House seats and $350M in the Senate. Democrats spent nearly identical amounts.

**Fig 2:**
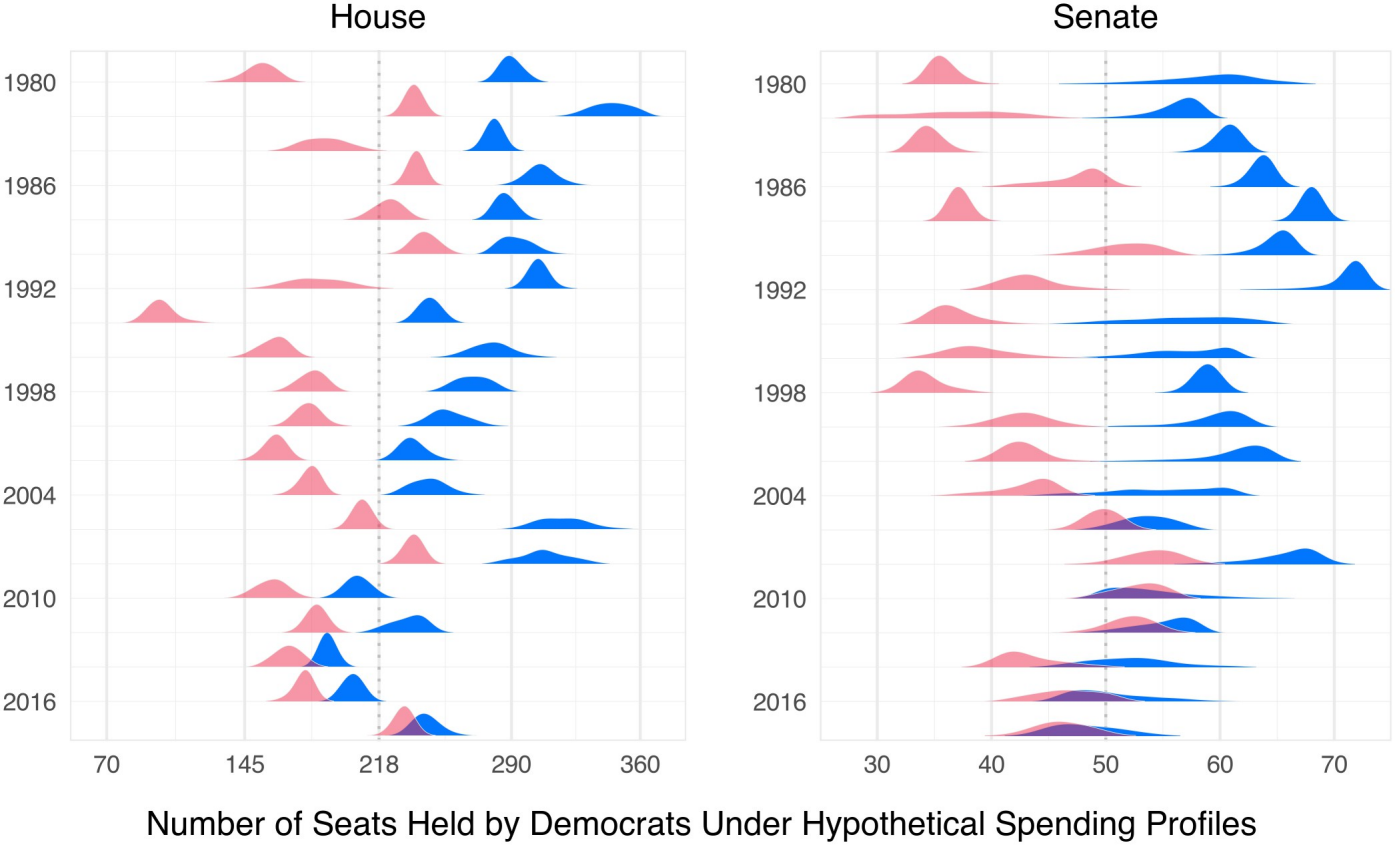
Congressional control depends on spending. Densities indicate simulated distributions of seats held by Democrats under hypotheticals with Democrats’ spending advantage held at the 95^th^ percentile (darker/blue) and at the 5^th^ percentile (lighter/red). The vertical dashed line indicates party control.

We count a seat as won by a Democrat if the estimated value of *Democratic Vote Share* exceeds 50%, or, when uncontested or otherwise excluded from our analysis, if the seat was ultimately held by a Democrat. Fig 2 displays results for both hypotheticals, depicting Democratic seats for each year-chamber. Darker/blue represents the hypothetical in which Democratic candidates spend more; lighter/red, the reverse. The dashed line separates Democratic majority from minority status.

The outcome illustrated in Fig 2 is striking. In all cases a positive number of seats change hands when the Democrats’ spending advantage changes from the 5^th^ to the 95^th^ percentile, in some cases as many as a third of a chamber. Whenever a pair of densities straddle the vertical line, it indicates that the majority party would switch from Republican to Democrat when the Democrats’ spending advantage changes from the 5^th^ to the 95^th^ percentile, so that control of Congress could be purchased. In the House, this would happen more than half of the time. In the Senate, control would switch in all but a handful of years. Even filibuster pivots would sometimes flip. Thus, it often appears possible to purchase control of Congress, under circumstances in which it would be possible to increase the Democrats’ spending advantage from the 5^th^ to the 95^th^ percentile.

**Fig 3:**
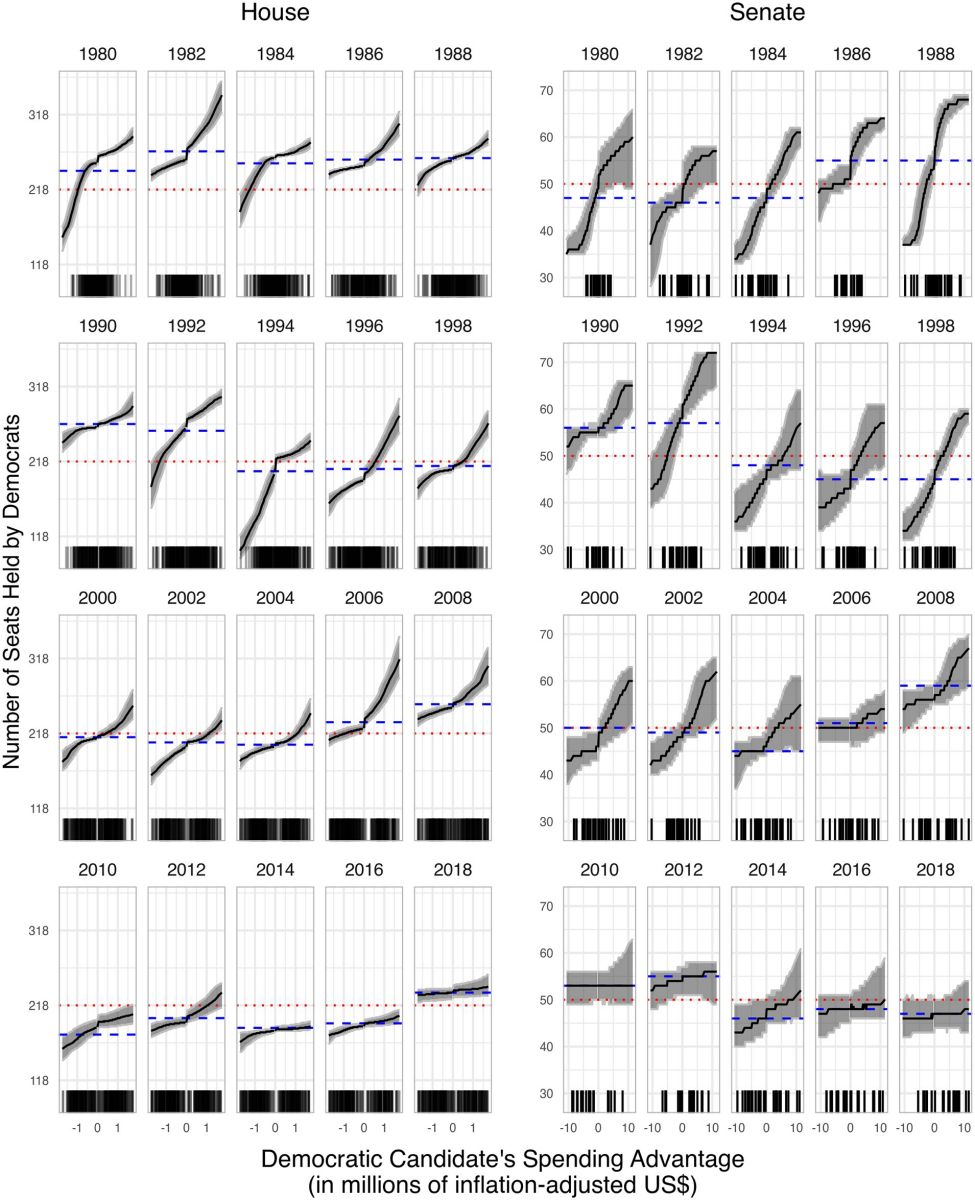
Congressional control with different spending advantages. The expected number of seats held by Democrats under hypotheticals that vary Democrats’ spending advantage from its largest value we consider (the 95^th^ percentile) to its smallest (the 5^th^ percentile) in increments of one percent. The red dotted lines indicate party control. The blue dashed lines indicate actual outcomes.

Next, in Fig 3, we show that our conclusion about purchasing control of Congress can remain true even when hypothetical spending advantages are reduced. In that figure, the left and right ends of each x-axis corresponds to Democrats’ spending advantage at the 5^th^ and the 95^th^ percentile, respectively—the values captured in Fig 2. In between those extremes, we repeat our analysis for less extreme counterfactuals. As one would expect, increasing the Democrats’ spending advantage tends to increase their expected number of seats held. The blue dashed line on each plot indicates the actual outcome, whereas the red dotted line indicates a switch of party control. Should the predicted number of seats held by Democrats cross that red line, party control has switched. If that happens for a less extreme hypothetical, then control of Congress can be purchased even when hypothetical spending advantages are reduces. As we can see from the figure, in many cases, party control flips for an amount of spending well below the 95^th^ percentile or above the 5^th^ percentile. Intriguingly, though, swings in seats also appear to be declining in recent years.

Of course, our findings in Figs 2 and 3 depend on the strong assumption that the opposing party does not match any additional expenditures. For that reason, our result ought to be interpreted as a partial equilibrium outcome. Yet, we stress that it is not clear that national parties always have the capability or willingness to match dollar for dollar—certainly not across all contests.

**Fig 4:**
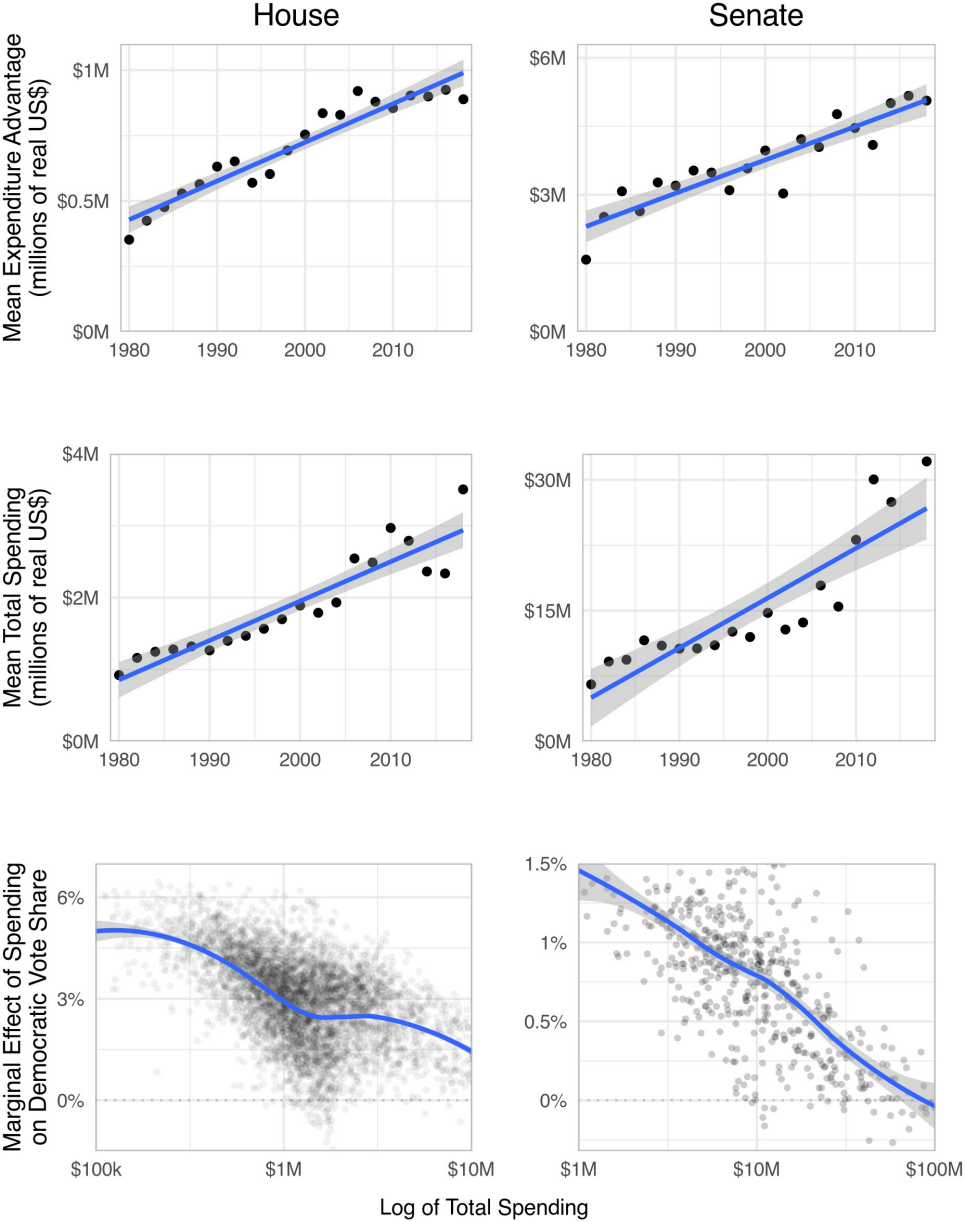
Increasing gaps and decreasing returns attenuate spending effects. Dots in the first two rows of panels represent average amounts spent by the larger spender minus those for the smaller spender, by year; lines are linear trends. The bottom row presents marginal effects from KRLS models, along with LOESS-smoothed averages.

We illustrate rising spending gaps in the top row of Fig 4, which illustrates the increasing gap in spending between the higher-spending front-runner, and the lower-spending underdog, and which suggests that matching is increasingly uncommon. These increasing gaps come as total spending has increased, as shown in the middle row of Fig 4. The bottom row of Fig 4, then, reveals that as more and more money is spent on campaigning, each additional dollar spent will generate additional returns in terms of vote share. The ability to match, and thus attenuate the large system-level effects we identified, is constrained by the availability of money to campaigns, which is in turn endogenous to total spending, campaign performance, the ideological landscape, the economy, campaign finance laws, and much else. Further changes in structural factors—potential campaign finance reforms [21], demographic trends, variation in donors’ preferences [22], or technological innovations in campaigning—could tilt the playing field and alter any general equilibrium result without altering our partial equilibrium outcomes. Understanding this complex system, and thereby establishing the most reasonable hypotheticals that would capture general equilibrium effects, awaits future work.

**Fig 5:**
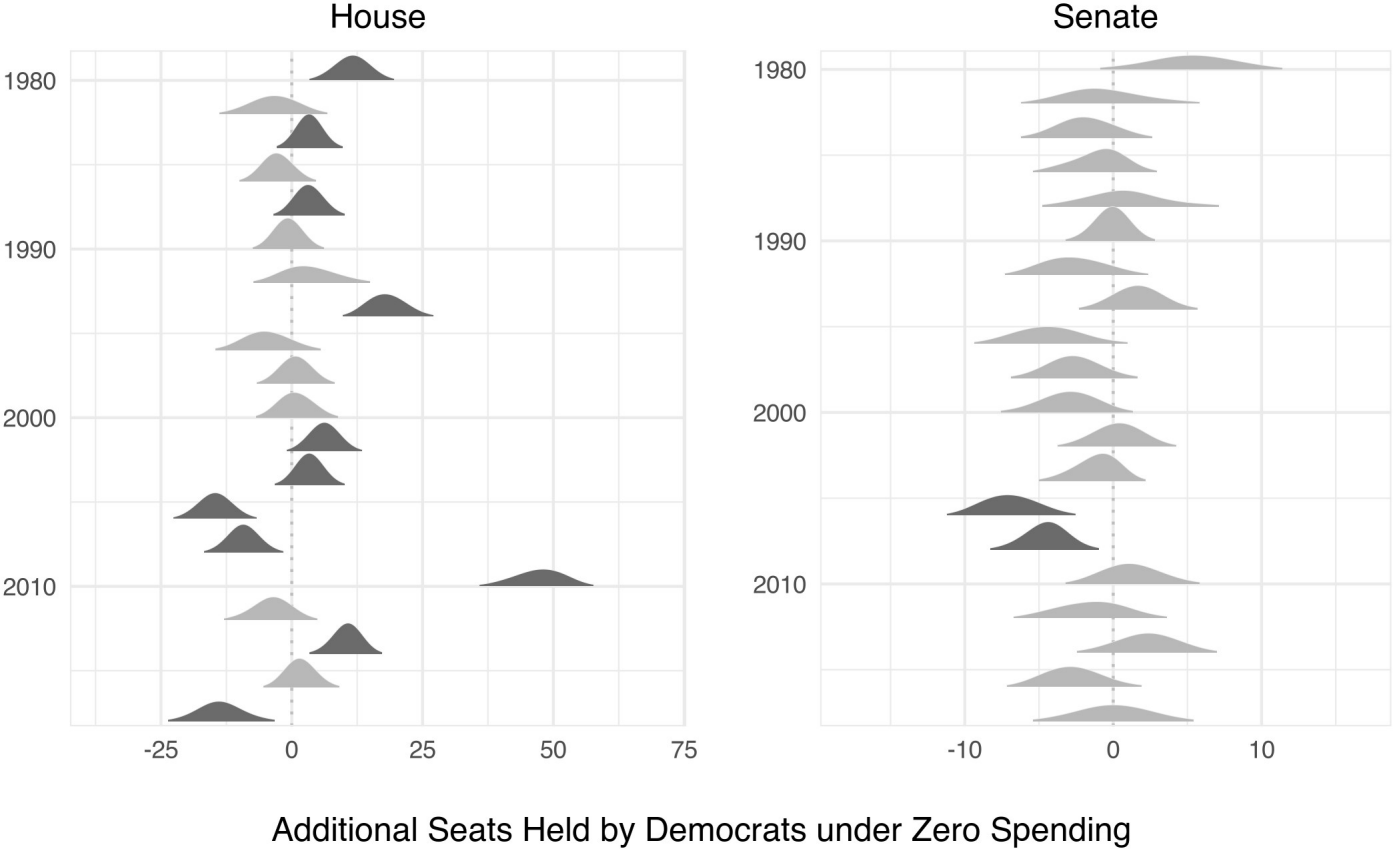
The effect of removing money on Democratic seats. Densities indicate simulated distributions of the difference between numbers of seats held by Democrats under the hypothetical zero-spending case minus that under the actually observed case. Darker gray densities indicate cases in which the 95% interval excludes zero.

We now turn to estimating the effects of mostly eliminating spending. The simulations displayed in Fig 5 compare outcomes with actual spending levels to those with a schedule that zeros out all expenditure advantages in contested races. The model differentiates between spending parity at zero and parity at other levels insofar as it includes log *Total Expenditures*, which we set at their minimum observed levels for our “zero”-spending hypothetical. The minimum observed levels were $33K in the Houseand $735K in the Senate. A positive seat swing indicates a situation in which eliminating expenditures increases the number of Democrat-held seats compared to actual expenditures. Darker densities indicate when zero is excluded from the 95% interval.

Three results emerge. First, electoral distortions due to spending appear in several cases—about half the time in the House, more rarely in the Senate. Second, eliminating spending in House elections appears to help Democrats more than Republicans. Third, in contrast, in those cases when eliminating spending has significant effects in the Senate, it benefits Republicans.

An explanation of these last two findings awaits future work. But it seems clear that spending can have a substantial effect on electoral outcomes. To assess robustness, we replicated all analyses using support vector machine regression and the nonparametric bootstrap (see Appendix H, S1 File). Results are strikingly similar. In fact, KRLS appears more conservative, suggesting our results may be underestimates.

That said, Figs 2 and 3 do suggest an apparent decline in spending effects. We offer three possible explanations. First, increasingly gerrymandered districts could potentially blunt the effect of both parties’ candidates’ spending, as redistricting has generally decreased electoral competitiveness [23]. Second, American politics has nationalized and refocused around parties’ presidential politics, leading to a decline in the electoral advantage enjoyed by incumbents [24] and increased outside spending [25], which may contribute to declining marginal returns, as ever smaller populations are left to mobilize. Third, contests may be marked by rising absolute differences in expenditures, which, according to theory and Fig 1, would decrease marginal effects. Fig 4 supports this explanation: its top two rows indicate that both total spending and mean expenditure advantages are increasing similarly over time; its bottom row illustrates that increases in total spending lead to decreases in the marginal effect of spending, suggesting that the marginal effect of spending is decreasing over time.

The implications of our findings speak more broadly to the debate on the role and utility of electoral campaigns [26–28]. Fig 3 illustrates that, for at least some election years, money can be used to help swing congressional majorities in both the House and Senate. This suggests that the money being spent by congressional candidates for advertisements [29], door knocking [30], determining which voters are persuadable [31], and so on can have meaningful collective consequences for electoral success. It also suggests that differences in who has access to campaign receipts [32] and how money is spent [11] may drive electoral outcomes in important ways. Finally, our results are also complementary to the recent work of Peress [33] that assesses the marginal effect of control of redistricting on seats in Congress. Even if partisan redistricting were to have an effect of small magnitude, that effect could exacerbate the effect we find, if the same party benefited in both cases.

## Conclusion

How should campaign spending laws, regulations, and rules be designed? And, without randomized controlled trials, how can we best gauge the effects of campaign spending on American politics? Both questions are important, but our best attempts to answer the latter question rely on assumptions that hamper our ability to answer the former.

By estimating the effects of spending at the seat-level and then aggregating those effects up to the levels of the House and Senate, we have been able to provide the first estimates of campaign spending effects at the system-level. Accounting for theoretically expected nonlinearities, our seat-level estimates are substantial when spending is near parity, and positive for most seats. Scaling up, we simulated national congressional elections, estimating that, under some conditions, money *can* buy control of Congress. At the same time, we also show that those conditions appear to have become less common over the past decade: we predict that fewer changes in party control would occur across the broad range of the counterfactuals we examined. *Removing* money from the equation can also alter outcomes, resulting in significant projected changes in partisan composition of the House in roughly half of all elections between 1980 and 2018, suggesting that existing expenditure levels already affect the composition of Congress.

While the system-level effects we identify, in terms of partisan composition of Congress and even, potentially, of party control, can be large, they also seem to be declining, in that we predict party control would be less likely to change hands across a broad range of spending profiles over the past decade. We offered possible explanations for this decline, including increasing gerrymandering and declining marginal returns due to either increased outside spending or rising absolute differences in expenditures, and note in conclusion that those reasons are themselves not immutable. For example, the extent of gerrymandering might change with court rulings or supermajority state control, and expenditure differences might change with new laws, changing economic conditions, or improved candidate recruitment by parties. Our study is only the first step toward a total, system-level view of how campaign expenditures affect American politics.

## Supporting information

S1 File Supplementary appendices. (PDF)

## References

[1] DeSilver D, van Kessel P. As More Money Flows into Campaigns, Americans Worry about its Influence; 2015. Available from: http://pewrsr.ch/1OPPiVu.

[2] Ansolabehere S, De Figueiredo JM, Snyder JM. Why Is There So Little Money in US Politics? Journal of Economic Perspectives. 2003;17(1):105–30.

[3] Bonica A. Mapping the Ideological Marketplace. American Journal of Political Science. 2014;58(2):367–86. doi: 10.1111/ajps.12062

[4] Kolodny R, Dwyre D. Convergence or divergence? Do parties and outside groups spend on the same candidates, and does it matter? American Politics Research. 2018;46(3):375–401.

[5] Drutman L. Why Money Still Matters; 2012. Available from: http://themonkeycage.org/2012/11/why-money-still-matters/.

[6] Gerber AS. Does Campaign Spending Work? Field Experiments Provide Evidence and Suggest New Theory. American Behavioral Scientist. 2004;47(5):541–74. doi: 10.1177/0002764203260415

[7] Stratmann T. Some Talk: Money in Politics. A (Partial) Review of the Literature. Public Choice. 2005;124:135–56. doi: 10.1007/s11127-005-4750-3

[8] Milyo J. Money in Politics. In: Scott R, Kosslyn S, editors. Emerging Trends in the Social and Behavioral Sciences. Wiley; 2015. p. 1–9.

[9] Jacobson GC. How Do Campaigns Matter? Annual Review of Political Science. 2015;18:31–47. doi: 10.1146/annurev-polisci-072012-113556

[10] Sides J, Vavreck L, Warshaw C. The Effect of Television Advertising in United States Elections. American Political Science Review. 2022;116(2):702–718. doi: 10.1017/S000305542100112X

[11] Fowler EF, Franz MM, Martin GJ, Peskowitz Z, Ridout TN. Political Advertising Online and Offline. American Political Science Review. 2021;115(1):130–149. doi: 10.1017/S0003055420000696

[12] Jacobson GC. The Effects of Campaign Spending in Congressional Elections. American Political Science Review. 1978;72(2):469–91. doi: 10.2307/1954105

[13] Erikson RS, Palfrey TR. Equilibria in Campaign Spending Games: Theory and Data. American Political Science Review. 2000;94:595–609. doi: 10.2307/2585833

[14] Gerber AS. Estimating the Effect of Campaign Spending on Senate Election Outcomes Using Instrumental Variables. American Political Science Review. 1998;92(2):401–11. doi: 10.2307/2585672

[15] Skaperdas S, Grofman B. Modeling Negative Campaigning. American Political Science Review. 1995;89:49–61. doi: 10.2307/2083074

[16] Hainmueller J, Hazlett C. Kernel Regularized Least Squares: Reducing Misspecification Bias with a Flexible and Interpretable Machine Learning Approach. Political Analysis. 2013;22(2):143–68. doi: 10.1093/pan/mpt019

[17] Skaperdas S. Contest Success Functions. Economic Theory. 1996;7:283–90. doi: 10.1007/BF01213906

[18] Groseclose T, Snyder JM. Buying Supermajorities. American Political Science Review. 1996;90(2):303–15. doi: 10.2307/2082886

[19] McGhee E. National Tides and Local Results in US House Elections. British Journal of Political Science. 2008;38(4):719–38. doi: 10.1017/S0007123408000355

[20] McDonald MP. United States Elections Project; 2018. Available from: http://www.electproject.org/home/voter-turnout/voter-turnout-data.

[21] La Raja RJ, Schaffner BF. Campaign finance and political polarization: When purists prevail. University of Michigan Press; 2015.

[22] Li Z. How Internal Constraints Shape Interest Group Activities: Evidence from Access-Seeking PACs. American Political Science Review. 2018;112(4):792–808. doi: 10.1017/S0003055418000382

[23] Henderson JA, Hamel BT, Goldzimer AM. Gerrymandering Incumbency: Does Nonpartisan Redistricting Increase Electoral Competition? Journal of Politics. 2018,80(3):1011–1016. doi: 10.1086/697120

[24] Jacobson GC. It’s Nothing Personal: The Decline of the Incumbency Advantage in US House Elections Journal of Politics. 2015,77(3):861–873. doi: 10.1086/681670

[25] Jacobson GC. Driven to Extremes: Donald Trump’s Extraordinary Impact on the 2020 Elections Presidential Studies Quarterly. 2021,51(3):492–521.

[26] Hillygus DS. Campaign effects and the dynamics of turnout intention in election 2000. The Journal of Politics. 2005;67(1):50–68. doi: 10.1111/j.1468-2508.2005.00307.x

[27] Kalla JL, Broockman DE. The minimal persuasive effects of campaign contact in general elections: Evidence from 49 field experiments. American Political Science Review. 2018;112(1):148–166. doi: 10.1017/S0003055417000363

[28] Kahn KF, Kenney PJ. The spectacle of US Senate campaigns. Princeton University Press; 2021.

[29] Vavreck L, et al. The exaggerated effects of advertising on turnout: The dangers of self-reports. Quarterly Journal of Political Science. 2007;2(4):325–343.

[30] Green DP, Gerber AS. Get out the vote: How to increase voter turnout. Brookings Institution Press; 2019.

[31] Hillygus DS, Shields TG. The persuadable voter: Wedge issues in presidential campaigns. Princeton University Press; 2008.

[32] Sorensen A, Chen P. Identity in Campaign Finance and Elections: The Impact of Gender and Race on Money Raised in 2010–2018 US House Elections. Political Research Quarterly. 2021; p. 10659129211022846.

[33] Peress M, Zhao Y. How Many Seats in Congress Is Control of Redistricting Worth? Legislative Studies Quarterly. 2020;45(3):433–468. doi: 10.1111/lsq.12268

