# Supplementary material for: Can money buy control of Congress?
Source: PLoS One. 2024 Jun 26;19(6):e0305846. doi: 10.1371/journal.pone.0305846 (PMC11207044; doi:10.1371/journal.pone.0305846)
Supplement: S1 File — (PDF) [file pone.0305846.s001.pdf]

Supplementary Material for

Can Money Buy Control of Congress?

William Minozzi* Gabriel J. Madson† David A. Siegel‡

Contents

Appendix A: Aggregate Simulation Method **S1**

Appendix B: Dataset Construction **S4**

Appendix C: KRLS Model Description & Results **S7**

Appendix D: Descriptive Statistics **S10**

Appendix E: Out-of-sample Accuracy & Alternative Methods **S11**

Appendix F: KRLS Models with Lagged Outcomes **S14**

Appendix G: KRLS with Dichotomous Outcomes **S16**

Appendix H: Replication w/ SVM & Nonparametric Bootstrap **S18**

*Professor, Department of Political Science, The Ohio State University; E-mail: minozzi.1@osu.edu.

†Research Public Health Analyst II, RTI International; E-mail: gmadson@rti.org.

‡Professor, Department of Political Science and Public Policy, Duke University; E-mail: david.siegel@duke.edu.

Appendix A: Aggregate Simulation Method

To simulate hypotheticals, we used the KRLS models detailed in the paper and Appendix C. For each chamber, we used both the predicted means and estimated variance-covariance matrices for the set of contested seats, drawing 5000 samples from the multivariate normal distribution. We did so for four cases: (1) actual spending levels; (2) zero spending advantage, in which we held total spending at its minimum observed level for all seats; (3) cases in which the Democratic candidate spent more, in which we set Democratic Expenditure Advantage at a quantile larger than the median such that Democratic Expenditure Advantage was positive; and (4) cases in which the Republican candidate spent more, in which we set Democratic Expenditure Advantage at a quantile smaller than the median such that Democratic Expenditure Advantage was negative. Finally, we accounted for all seats that were either not up for reelection, uncontested, or dropped from the dataset for some other reason. We used these simulations to illustrate hypothetical control of Congress under different spending profiles.

In more detail, we used the R code below to produce these simulations. The code consists of a function that takes four arguments: (1) a type (a string "actual" or "zero") or quantile (a number from 0.05 to 0.95), (2) a fitted KRLS model (either that for the House or the Senate), (3) the dataset used to produce that model, and (4) the desired number of simulations, which defaults to 5000.

The function first checks whether the arguments are legal, and then defines three things: a new dataset of predictors `pred_X`, which is a copy of the predictors used in the KRLS model, the vector of actual observed values of Democratic Expenditure Advantage, and the vector of years corresponding to each of the contests included in the dataset. If the function is being used to simulate values at a quantile of Democratic Expenditure Advantage, that spending level is then calculated and called `simulated_dem_spending_advantage`, because we index the hypotheticals in the last case by the observed quantile of Democratic Expenditure Advantage, ranging from the 5th percentile (a large Republican expenditure advantage) to the 95th percentile (a large Democratic expenditure advantage).

Exactly what happens next depends on the type or quantile called for, but regardless of which of these hypotheticals is desired, the function will create the appropriate value of predictors (i.e., `pred_X`). When "actual" is called, `pred_X` is not altered at all. When "zero" is called, the column of `pred_X` which stores hypothetical values of Democratic Expenditure Advantage is set equal to 0, and the column of `pred_X` which stores hypothetical values of log Total Expenditure is set equal to its minimum observed value.

For any hypothetical quantile value $q \in [0.05, 0.95]$, `pred_X` is altered in keeping with a scenario in which (for all races in the middle 90% of the Democratic Expenditure Advantage distribution) one of the two candidates is "topped up."

To elaborate, for any positive value of `simulated_dem_spending_advantage`, we sweep through contests in the middle 90% of Democratic Expenditure Advantage, identifying those in which the Democratic candidate did not outspend her opponent at the desired level. We set the value of Democratic Expenditure Advantage to `simulated_dem_spending_advantage` in those rows, and change the value of log Total Expenditures to the appropriate corresponding value that would result from such additional spending. In other words, for all contests not in the tails of the spending distribution, we check to see if the Democratic candidate had a spending advantage less than the amount specified in the hypothetical, and increase their spending advantage to that point if so. That is what we mean above by "topped up."

We do a similar process when `simulated_dem_spending_advantage` < 0, which corresponds to hypothetical scenarios in which all Republican candidates outspend their Democratic rivals. Specifically, for all contests in the middle 90% of Democratic Expenditure Advantage, we check to see if the Democratic candidate had a spending advantage greater than the amount specified in the hypothetical, and decrease their spending advantage to that point if so.

Given the completed `pred_X` object, we produce simulated values of Democratic Vote Share in all races by drawing from a multivariate Normal distribution with mean equal to the predicted values from the the fitted KRLS model evaluated at `pred_X` and the variance-covariance matrix provided by KRLS. It is important to draw all races from one multivariate Normal distribution because different observations will covary with each other. We draw 5000 such simulations for the vector of Democratic Vote Share values.

Finally, for each simulation and each contest, we record whether Democratic Vote Share was above 50%, and if so, count that as a Democratic victory in that race. We then sum the number of Democratic victories for each year and simulation. The function returns a data.frame containing simulated numbers of seats held by Democrats and Republicans, for each year and each simulation, as well as the type or quantile called for by the function.

After running this function, we merge in the observed outcomes of races that were dropped from the dataset, e.g., unopposed candidates. The result is a set of 5000 simulated outcomes for each chamber, each year, and each hypothetical expenditure advantage.

The paper reports on simulations for "actual", "zero", maximum Democratic Expenditure Advantage (quantile = 0.95), and maximum Republican Expenditure Advantage (quantile = 0.05), as well as quantile values between 0.05 and 0.95, with increments of 0.01.

```
make_simulations <- function(
  type_or_quantile, # number in [0.05, 0.95], or string "actual" or "zero"
  krls_model,       # the fitted KRLS model
  data,             # dataset used to fit KRLS model
  n_boots = 5000    # number of simulations to draw
  ) {
  # these are the only safe simulations from the model
  stopifnot(
    type_or_quantile %in% c("zero", "actual") |
     (type_or_quantile >= .05 & type_or_quantile <= .95))
  pred_X <- copy(krls_model$X)
  actual_dem_spending_advantage <-
    pred_X[, "dem_spend_adv"] +
    pred_X[, "bottom_tail_DSA"] +
    pred_X[, "top_tail_DSA"]
  year <-
    1980 * pred_X[, "y80"] + 1982 * pred_X[, "y82"] +
    1984 * pred_X[, "y84"] + 1986 * pred_X[, "y86"] +
    1988 * pred_X[, "y88"] + 1990 * pred_X[, "y90"] +
    1992 * pred_X[, "y92"] + 1994 * pred_X[, "y94"] +
    1996 * pred_X[, "y96"] + 1998 * pred_X[, "y98"] +
    2000 * pred_X[, "y00"] + 2002 * pred_X[, "y02"] +
    2004 * pred_X[, "y04"] + 2006 * pred_X[, "y06"] +
    2008 * pred_X[, "y08"] + 2010 * pred_X[, "y10"] +
    2012 * pred_X[, "y12"] + 2014 * pred_X[, "y14"] +
    2016 * pred_X[, "y16"] + 2018 * pred_X[, "y18"]
  if (!is.na(as.numeric(type_or_quantile))) {
    simulated_dem_spending_advantage <-
      quantile(actual_dem_spending_advantage, type_or_quantile)
  }
  if (type_or_quantile == "actual") {
  } else if (type_or_quantile == "zero") {
    pred_X[, "dem_spend_adv"] <- 0
    pred_X[, "log_total_spending"] <- min(pred_X[, "log_total_spending"])
  } else if (simulated_dem_spending_advantage > 0) {
    rows_to_change <- which(
      pred_X[, "bottom_tail"] + pred_X[, "top_tail"] == 0 &
        actual_dem_spending_advantage < simulated_dem_spending_advantage)
    pred_X[rows_to_change, "dem_spend_adv"] <-
      simulated_dem_spending_advantage
    pred_X[rows_to_change, "log_total_spending"] <- log10(
      2 * data[rows_to_change, real_rep_expenditure_w_outside] +
        abs(simulated_dem_spending_advantage) * 1e6)
  } else if (simulated_dem_spending_advantage < 0) {
    # include .5 here because median obs has R spend more than D; i.e. DSA < 0
    simulated_dem_spending_advantage <-
      quantile(actual_dem_spending_advantage, type_or_quantile)
    rows_to_change <- which(
      pred_X[, "bottom_tail"] + pred_X[, "top_tail"] == 0 &
        actual_dem_spending_advantage > simulated_dem_spending_advantage)
    pred_X[rows_to_change, "dem_spend_adv"] <-
      simulated_dem_spending_advantage
    pred_X[rows_to_change, "log_total_spending"] <- log10(
      2 * data[rows_to_change, real_dem_expenditure_w_outside] +
        abs(simulated_dem_spending_advantage) * 1e6)
  }
  pred <- predict(krls_model, newdata = pred_X, se.pred = TRUE)
  sims <- MASS::mvrnorm(n_boots, pred$predicted,
    as.matrix(pred$vcov.est.pred))
  simulations <- CJ(index = 1:nrow(pred_X), boot = 1:n_boots)
  simulations[, `:=`(
    simulated_dem_spend_adv =
      (pred_X[, "dem_spend_adv"] +
         pred_X[, "bottom_tail_DSA"] +
         pred_X[, "top_tail_DSA"])[index],
    actual_dem_spending_advantage = actual_dem_spending_advantage[index],
    boot_y = as.vector(sims),
    year = year[index],
    type_or_quantile = type_or_quantile,
    nonoutlier = (1 - pred_X[, "bottom_tail"] - pred_X[, "top_tail"])[index]
  )]
  simulations[, party_of_winner := ifelse(boot_y > .5, "D", "R")]
  return(simulations[, .(
    n_dems = sum(boot_y > .5),
    n_reps = sum(boot_y < .5)), .(
      type_or_quantile, year, boot)])
}
```

Appendix B: Dataset Construction

Our datasets comprise all House and Senate contests in the 19 general elections from 1980 to 2018. In the House, we have $8700 (= 20 \times 435)$ observations, and in the Senate, 667. For comparability, we exclude off-cycle Senate elections for the balances of incomplete terms that were held on general election dates.

For both House and Senate datasets, we started with the "Statistics of the Presidential and Congressional Election" compiled and published by the clerk of the House after each election, from which we gathered all candidates in each contest. We isolated those cases in which there was one Democratic party candidate and one Republican party candidate, and identified their names and vote totals.

These data were then merged with selected columns from Gary Jacobson's dataset on quality challengers and Adam Bonica's Database on Ideology, Money, and Elections (DIME), matching states, districts (for the House), election cycles, and names, cleaning where necessary. In addition to challenger quality measures, Jacobson's data provided indicators for whether a seat was open, included a Democratic party incumbent, occurred immediately after redistricting, or included some other event that rendered the contest noncomparable with contests that included two opposing major party candidates. Jacobson also provides presidential vote share at the House district level. These totals are from the most recent election where possible (e.g., mid-decade, midterm elections), from concurrent results for presidential election years, adjusting for redistricting where necessary. Results are similar when we drop House races with contemporaneous presidential elections. In the Senate, we use statewide Democratic presidential vote share from the most recent election. DIME provided matches to both Bonica's measure of ideology (CF scores) and FEC identifiers. We rely on CF scores, which are measures of candidate ideology based on the giving patterns of political donors, because it allows us to obtain ideological point estimates for challengers. Alternative methods for obtaining candidate ideology, like DW-NOMINATE, only provide ideology estimates for incumbents, which is why we instead rely on CF scores. The value of combining evidence from original sources and those collected separately by Jacobson and Bonica is that we could triangulate any discrepancies.

More generally, we include variables such as ideology and state population for several reasons. One is that each had been used in prior literature on understanding vote totals. For example, Abramowitz (1988) for state populations in Senate races and Ensley (2009) for ideology difference. A second is to account for potential bang-for-the-buck. Larger populations might suggest a lower marginal effect of money. Or the marginal effect of money might be different for those closer to a district median and for those further away, perhaps because money helps candidates become better known. A third is to capture other possible connections between those variables and the effect of money on elections. For instance, it is possible that ideology influences available money, and that available money helps determine expenditures. One could imagine other scenarios as well. Because KRLS allows the model specification to be, in a sense, determined by the data, by including both expenditures and ideology, we allow the model to account for any influence if such exists.

Given the aforementioned contest and candidate identifiers, we next merged in the relevant expenditure variables from the "all candidates file". Specifically, we selected the following columns: "Candidate identification," "Candidate name," "Party affiliation," "Candidate state" and "Candidate district," "Primary election status" and "General election status," and finally "Total

disbursements." We used all but the last column to identify total disbursements for all major candidates in the universe of contests, repairing missing or incorrect observations where necessary. For the Senate contests, we also brought in the log of each state's Voter Eligible Population for a given election year – relying on data from McDonald's *United States Elections Project.* This was done to control for the possibility of larger populations having a lower marginal effect of money.

We further used the available sources to identify events that rendered races noncomparable. Given these cleaned major party candidates, we next added in major independent candidates who caucused with a major party (e.g., Bernie Sanders, Virgil Goode, etc.). For example, we identified all contests from Louisiana in which the jungle primary included more than one major party candidate from a single party. In those cases, we replaced the row with the runoff election where possible. To accommodate cases in which a candidate won the jungle primary outright despite being one of several major party candidates, we created a variable named "Jungle" which we later used to exclude that row from analysis. Similarly, we identified other odd cases, including contests with two major party candidates from the same party because of a top two primary, or those with only one major party candidate and an independent who had not yet caucused with the opposing party.

Once we identified all major party candidates for all House and Senate races, we gathered, cleaned, and merged in outside spending by interest groups. Specifically, we gathered all spending on "electioneering communications",[1] "communication costs",[2] "party coordinated expenditures",[3], and "independent expenditures".[4] These data sources include indicators for spending by outside groups that include reference to specific candidates. In many cases, these outside groups are considered to be "dark money" because they do not disclose their donors; they are included, however, whenever they report spending money in particular contests. Indeed, many congressional contests attract considerable amounts of dark money, and that spending is transparent even though the donors to those groups remain unknown.[5] We next cleaned these data, repairing mangled identification numbers, candidate names, geographic identifiers, etc. For the last category, records also included indicators for whether the group supported or opposed the candidate. We further coded party coordinated expenditures to indicate support for a candidate. For the first two categories, we had to supply such indicators on a case-by-case basis. To do so, we coded interest groups as conservative or liberal where possible, based on evidence from opensecrets.org. Such coding was not possible in the case of major trade associations that sometimes campaigned on behalf of candidates from both parties, but the vast majority of spending was easily identified using this method. Based on this strategy, we measured outside spending for and opposed to each candidate.

Importantly, outside spending changed dramatically during our study period, due to changes in law including the Bipartisan Campaign Reform Act and *Citizens United.* Consequently, we re-estimated our models on subsets of our dataset. The conclusions presented in the paper appear

[1]https://www.fec.gov/data/electioneering-communications/, last retrieved 2020-08-26.

[2]https://www.fec.gov/data/communication-costs/, last retrieved 2020-08-26.

[3]https://www.fec.gov/data/party-coordinated-expenditures/, last retrieved 2020-08-26.

[4]https://www.fec.gov/data/independent-expenditures/, last retrieved 2020-08-26.

[5]See https://www.opensecrets.org/dark-money/top-elections, last retrieved 2020-08-26.

not to change dramatically based on these inclusion criteria.

Appendix C: KRLS Model Description & Results

Our key measure of *Democratic Expenditure Advantage* is an interaction term that is held at zero for observations in the top 5% and bottom 5% of its distribution. The reasons are (1) the distributions of *Democratic Expenditure Advantage* are both leptokurtic, meaning that they have fat tails and extreme outliers, and (2) inferences based on these tail values are unlikely to extrapolate well to the bulk of distribution. In the Senate, the middle 90% of the spending distribution ranges from $-\$10.5$M to $\$11.4$M, but the total range is from $-\$49$M to $\$78$M. Its excess kurtosis is 17.8. Similarly, in the House, the middle 90% of the spending distribution ranges from $-\$1.7$M to $\$1.7$M, but the total range is from $-\$24$M to $\$20$M. Its excess kurtosis is 43.7.

The observations in the tails may be misleading for several reasons. First, there are likely decreasing returns to scale from campaign spending. Second, values in the tails may often emerge from quixotic candidacies launched by wealthy candidates who go on to lose dramatically. Third, our hypotheticals of interest do not include the possibility for such gigantic investments in all races; instead, we are interested in the much more plausible ranges indicated by the middle 90% of the distribution.

We fit all KRLS models using the bigKRLS package (Mohanty and Shaffer, 2019) in R.

Figure S1 illustrates the same results as in the text's Figure 1, save without separate lines for incumbents so as to make comparisons to later figures in this appendix easier.

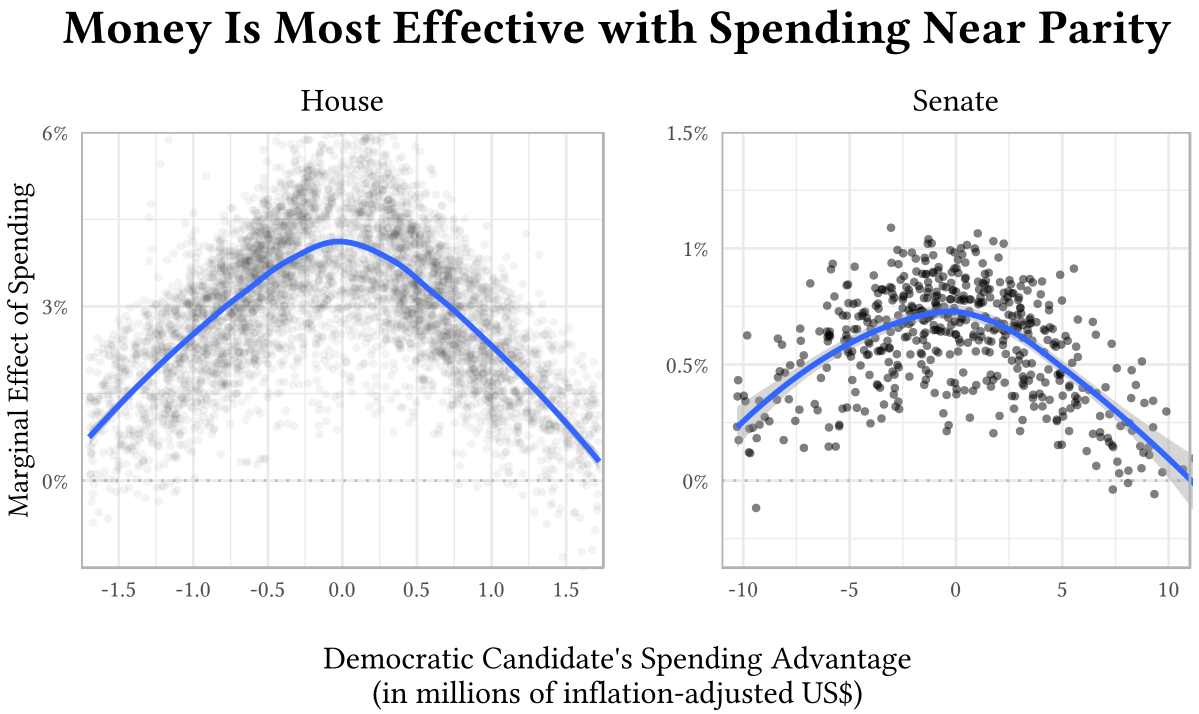

Figure S1: Points are estimated marginal effects of *Democratic Spending Advantage* based on KRLS models. Lines are LOESS fits.

Table S1: House KRLS Average Marginal Effects

Variable	Estimate	SE	p
Democratic Spending Advantage	0.0277	0.0011	< 0.001
log(Total Spending)	0.0003	0.0021	0.897
Democratic Presidential Vote Advantage	0.0043	0.0001	< 0.001
Ideological Distance	0.1437	0.0127	< 0.001
Democrat Candidate's CF Score	0.0068	0.0016	< 0.001
Republican Candidate's CF Score	0.0064	0.0019	< 0.001
Dem. Inc./Low Qual. Chall.	0.0584	0.0017	< 0.001
Dem. Inc./High Qual. Chall.	0.0433	0.0025	< 0.001
Rep Inc./Low Qual. Chall.	−0.0570	0.0016	< 0.001
Rep Inc./High Qual. Chall.	−0.0405	0.0024	< 0.001
Open Seat/Both High Qual.	−0.0059	0.0035	0.095
Open Seat/High Qual. Dem/Low Qual. Rep	0.0279	0.0039	< 0.001
Open Seat/Low Qual. Dem/High Qual. Rep	−0.0326	0.0039	< 0.001
Year = 1980	−0.0098	0.0028	< 0.001
Year = 1982	0.0175	0.0026	< 0.001
Year = 1984	−0.0119	0.0027	< 0.001
Year = 1986	0.0088	0.0026	< 0.001
Year = 1988	−0.0018	0.0026	0.491
Year = 1990	0.0064	0.0027	0.018
Year = 1992	0.0032	0.0024	0.183
Year = 1994	−0.0245	0.0024	< 0.001
Year = 1996	0.0034	0.0023	0.145
Year = 1998	0.0034	0.0027	0.214
Year = 2000	0.0011	0.0026	0.668
Year = 2002	−0.0068	0.0027	0.013
Year = 2004	0.0014	0.0025	0.574
Year = 2006	0.0189	0.0026	< 0.001
Year = 2008	0.0170	0.0026	< 0.001
Year = 2010	−0.0212	0.0025	< 0.001
Year = 2012	0.0082	0.0026	0.002
Year = 2014	−0.0128	0.0027	< 0.001
Year = 2016	−0.0038	0.0030	0.215
Year = 2018	0.0067	0.0030	0.026
Bottom 5%	−0.0287	0.0026	< 0.001
Middle 90%	0.0010	0.0017	0.560
Top 5%	0.0270	0.0026	< 0.001
Bottom 5% × Dem. Spending Adv.	0.0037	0.0002	< 0.001
Top 5% × Dem. Spending Adv.	0.0040	0.0002	< 0.001

$n = 5859$.

Table S2: Senate KRLS Average Marginal Effects

Variable	Estimate	SE	p
Democratic Spending Advantage	0.0044	0.0005	< 0.001
log(Total Spending)	0.0063	0.0054	0.244
Democratic Presidential Vote Advantage	0.0015	0.0001	< 0.001
Adj. Dem. Pres. Vote Advantage	0.0024	0.0002	< 0.001
log(n Votes for Democratic Presidential Candidate)	0.0065	0.0018	< 0.001
log(n Votes for Republican Presidential Candidate)	−0.0038	0.0024	0.104
Ideological Distance	0.1440	0.0323	< 0.001
Democrat Candidate's CF Score	0.0198	0.0048	< 0.001
Republican Candidate's CF Score	0.0148	0.0056	0.008
Open Seat	0.0160	0.0067	0.018
Democratic Incumbent	0.0834	0.0062	< 0.001
log(Voting Eligible Population)	0.0005	0.0019	0.789
Year = 1980	−0.0161	0.0081	0.049
Year = 1982	0.0215	0.0078	0.006
Year = 1984	−0.0101	0.0085	0.234
Year = 1986	0.0149	0.0100	0.137
Year = 1988	0.0196	0.0080	0.014
Year = 1990	0.0067	0.0092	0.467
Year = 1992	0.0095	0.0072	0.186
Year = 1994	−0.0211	0.0074	0.005
Year = 1996	−0.0052	0.0080	0.517
Year = 1998	−0.0024	0.0076	0.756
Year = 2000	−0.0020	0.0077	0.792
Year = 2002	−0.0038	0.0080	0.637
Year = 2004	0.0078	0.0081	0.332
Year = 2006	0.0286	0.0082	< 0.001
Year = 2008	0.0120	0.0080	0.137
Year = 2010	−0.0279	0.0085	0.001
Year = 2012	0.0092	0.0088	0.295
Year = 2014	−0.0200	0.0078	0.011
Year = 2016	−0.0111	0.0099	0.260
Year = 2018	0.0016	0.0096	0.870
Bottom 5%	−0.0123	0.0058	0.034
Middle 90%	−0.0035	0.0038	0.361
Top 5%	0.0190	0.0057	< 0.001
Bottom 5% × Dem. Spending Adv.	0.0002	0.0001	0.072
Top 5% × Dem. Spending Adv.	0.0004	0.0001	< 0.001

$n = 586$.

Appendix D: Descriptive Statistics

Table S3: House Summary Statistics

Variable	Mean	SD	Min	Max	# Missing
Democratic Vote Share	0.52	0.18	0.09	0.97	1207
Democratic Expenditure Advantage (Millions of US$)	−0.01	1.45	−24.03	20.33	2561
(log) Total Expenditure (Millions of US$)	6.15	0.36	4.52	7.81	2561
Democrat CF Score	−0.74	0.49	−4.33	2.25	953
Republican CF Score	0.89	0.39	−2.50	4.31	1455
Ideological Distance	0.01	0.06	−0.36	0.37	2376
Adjusted Democratic Presidential Vote Advantage	0.68	13.91	−33.65	54.25	1
Open Seat	0.10	0.31	0	1	0
Democratic Incumbent	0.47	0.50	0	1	1
Quality Challenger	0.15	0.35	0	1	1
Unopposed	0.24	0.43	0	1	1

Num. Obs. = 8700. Num. Complete Cases = 5859.

Table S4: Senate Summary Statistics

Variable	Mean	SD	Min	Max	Missing
Democratic Vote Share	0.50	0.13	0.12	0.85	24
Democratic Expenditure Advantage (Millions of US$)	−0.01	8.77	−48.96	78.21	75
(log) Total Expenditure (Millions of US$)	7.02	0.41	5.87	8.50	75
Democrat CF Score	−0.76	0.39	−2.29	0.77	34
Republican CF Score	0.88	0.31	−0.20	2.45	26
Ideological Distance	0.01	0.05	−0.12	0.19	56
Adjusted Democratic Presidential Vote Advantage	−2.10	8.08	−26.82	19.74	4
Voting Eligible Population (in millions)	2.89	3.56	0.16	28.17	0
Open Seat	0.19	0.40	0	1	0
Democratic Incumbent	0.42	0.49	0	1	0
Jungle Primary	0.01	0.08	0	1	0
Top Two General Election/Other Candidate/Etc.	0.01	0.10	0	1	0
Unopposed	0.04	0.19	0	1	0

Total Num. Obs. = 667. Num. Complete Cases = 586.

Appendix E: Out-of-sample Accuracy & Alternative Methods

For robustness, we also fit models using LASSO, support vector machine (SVM) regression, and random forest (RF) regression. While SVM and RF are nonparametric, the LASSO suffers from the same issues as linear models. In particular, without including the right slate of multiplicative interaction terms that interact *Democratic Expenditure Advantage* with other covariates, LASSO does not capture the nonlinearities we identify.[6] Because SVM and RF are nonparametric, they, like KRLS, accommodate these interactions automatically.

To probe the out-of-sample predictive accuracy of all four methods, Table S5 reports root mean squared error and R^2 from five-fold cross validation. In general, all four methods performed competitively, although RF offers the best predictions.

Table S5: Out-of-sample Predictive Accuracy

Mean Squared Error	House	Senate
KRLS	0.057	0.072
RF	0.049	0.059
SVM	0.049	0.071
LASSO	0.064	0.076
R^2	House	Senate
KRLS	0.871	0.686
RF	0.905	0.789
SVM	0.902	0.693
LASSO	0.836	0.640

One of the appeals of KRLS is that it also provides a direct model of heterogeneity in marginal effects. Because LASSO is a linear model, the estimated average marginal effect must be constant (absent interactions) and is simply the coefficient on *Democratic Expenditure Advantage*. The other approaches we use do not model these effects at all. Therefore, to probe the robustness of KRLS, we needed to estimate numerical derivatives for each of the other two methods.

Specifically, for SVM and RF, we (1) choose a small step size (root-one ten millionth of the largest observed magnitude of *Democratic Expenditure Advantage* within the middle 90% of its distribution), (2) use each method to predict outcomes from a step above and below the observed level for each observation, (3) and calculate the difference between these predictions, divided by twice the step size. Results appear in Table S6.

[6] We briefly consider adding higher-order polynomial terms below.

Table S6: Comparison of Average Marginal Effects Estimates

House	Estimate	SE	p
KRLS	0.030	< 0.001	< 0.001
RF	0.096	0.007	< 0.001
SVM	0.047	< 0.001	< 0.001
LASSO	0.022	< 0.001	< 0.001
Senate			
KRLS	0.006	< 0.001	< 0.001
RF	0.013	0.0005	0.012
SVM	0.006	< 0.001	< 0.001
LASSO	0.004	< 0.001	< 0.001

At the aggregate level, estimates are statistically significant in every case. As noted above, the LASSO is linear, and just like as with any linear model would be absent interaction effects without an *a priori* selection of the proper interaction terms. It is likely therefore depressed by smaller marginal effects in the tails. Of the three nonparametric methods, KRLS produced the smallest estimates of average marginal effects in both chambers. To the extent that the results in the paper are biased because of choice of learning method, this bias seems plausibly conservative.

To replicate Figure S1, we plot the estimated conditional average marginal effects in appendix Figure S2. Because the LASSO is linear, its prediction is flat, and we therefore omit it from the figure. Of the three nonparametric methods, KRLS is the most conservative, although its estimates are very similar to those from SVM. SVM also yields plausible marginal effects, with a range somewhat larger than those from KRLS, especially in the House. In contrast, the estimates from random forests are often implausible, with average marginal effects ranging from three to five times as large as those from KRLS and SVM. Further, the marginal effects ranges for random forests are extreme—between -170% and 2103% in the House, and between -38% and 186% in the Senate—which takes estimates several orders of magnitude outside of the logically possible range of the outcome variable.

Based on these comparisons, we are most confident about the estimates based on KRLS, which we document in the main paper, because they offer competitive out-of-sample predictive accuracy, produce plausible estimates of marginal effects, and are also the most conservative of the three nonparametric methods. LASSO likely performs the worst in terms of out-of-sample predictive accuracy because it does not accommodate heterogeneity in marginal effects without explicitly specifying interaction terms.[7] While RF offers the best out-of-sample predictive accuracy,

[7]Nevertheless, we further explored LASSO models, including the fourth-order polynomials in *Democratic Expenditure Advantage*. In the Senate, out-of-sample RMSE fell to 0.074 and out-of-sample R^2 rose to 0.66, while in the House, out-of-sample RMSE fell to 0.062 but out-of-sample R^2 remained at 0.84. These performance levels remained the worse performing of the four methods, although we re-emphasize that all four methods are competitive. One possibility for this is again that LASSO does not include interaction terms without prior specification, whereas the nonparametric methods permit higher order dependencies.

our method of calculating marginal effects based on RF yielded wildly implausible estimates.[8]

Finally, because SVM is competitive with KRLS on both plausibility and predictive accuracy, we use it in Supplemental Appendix Section I as a robustness check, replicating our analysis completely with this method.

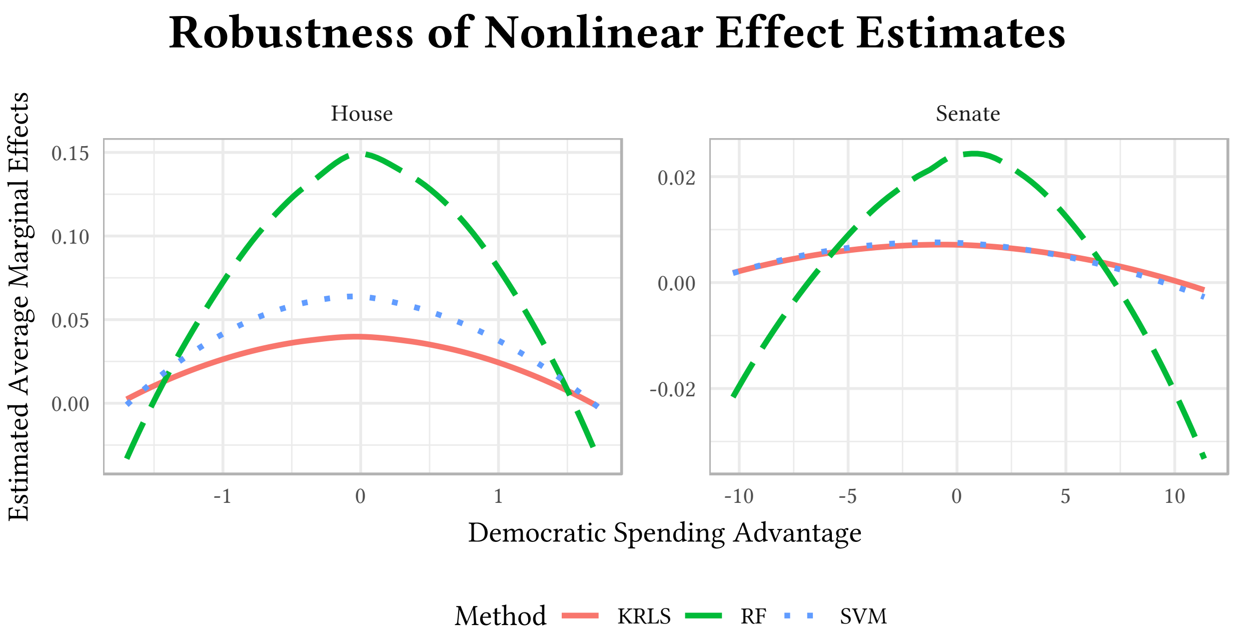

Figure S2: Replication of Figure S1 with alternative methods. The nonlinear finding from KRLS is replicated by both support vector machine regression and random forest regression.

[8]If you drop the marginal effects that are larger than 100% in magnitude, random forests do yield similar estimates to the ones we show in the paper. However, as dropping those effect sizes would be somewhat ad hoc, we preferred not to place our confidence in random forests in our application.

Appendix F: KRLS Models with Lagged Outcomes

In this section, we replicate our analyses, now adjusting for the lagged value of *Democratic Vote Share* at the seat or district level. Doing so necessitates that we drop observations where these values are not measurable, including our first cycle (1980) in the House and first three (1980, 1982, 1984) in the Senate, each House redistricting cycle (1982, 1992, 2002), any mid-decade redistricting, and all open seat races. Even after dropping these observations, estimates of average marginal effects from the KRLS models hew closely to the results presented in the paper. Average marginal effects are smaller in both cases, but still highly significant. We also explored models adjusting for lagged spending, and found similar results (average marginal effects of 0.0257 for the House and 0.0039 for the Senate, both with $p < 0.001$).

Table S7: House KRLS Average Marginal Effects, Incl. Lagged Outcome

Variable	Estimate	SE	p
Democratic Spending Advantage	0.0257	0.0013	< 0.001
log(Total Spending)	0.0101	0.0024	< 0.001
Democratic Presidential Vote Advantage	0.0030	0.0001	< 0.001
Ideological Distance	0.0662	0.0170	< 0.001
Democrat Candidate's CF Score	0.0041	0.0020	0.038
Republican Candidate's CF Score	0.0038	0.0024	0.111
Dem. Inc./Low Qual. Chall.	0.0338	0.0032	< 0.001
Dem. Inc./High Qual. Chall.	0.0349	0.0035	< 0.001
Rep Inc./Low Qual. Chall.	−0.0350	0.0029	< 0.001
Rep Inc./High Qual. Chall.	−0.0291	0.0033	< 0.001
Year = 1984	−0.0124	0.0029	< 0.001
Year = 1986	0.0140	0.0026	< 0.001
Year = 1988	−0.0001	0.0025	0.963
Year = 1990	0.0090	0.0027	< 0.001
Year = 1994	−0.0275	0.0023	< 0.001
Year = 1996	0.0110	0.0023	< 0.001
Year = 1998	0.0068	0.0025	0.007
Year = 2000	0.0041	0.0025	0.103
Year = 2004	0.0040	0.0025	0.104
Year = 2006	0.0215	0.0025	< 0.001
Year = 2008	0.0144	0.0025	< 0.001
Year = 2010	−0.0281	0.0024	< 0.001
Year = 2014	−0.0168	0.0026	< 0.001
Year = 2016	−0.0029	0.0033	0.383
Year = 2018	0.0124	0.0032	< 0.001
Bottom 5%	−0.0219	0.0034	< 0.001
Top 5%	0.0214	0.0034	< 0.001
Middle 90%	0.0005	0.0022	0.819
Bottom 5% × Dem. Spending Adv.	0.0041	0.0003	< 0.001
Top 5% × Dem. Spending Adv.	0.0034	0.0003	< 0.001
Lagged Outcome	0.2448	0.0081	< 0.001

$n = 3766$.

Table S8: Senate KRLS Average Marginal Effects, Incl. Lagged Outcome

Variable	Estimate	SE	p
Democratic Spending Advantage	0.0047	0.0004	< 0.001
log(Total Spending)	0.0147	0.0051	0.004
Democratic Presidential Vote Advantage	0.0013	0.0001	< 0.001
Adjusted Democratic Presidential Vote Advantage	0.0021	0.0002	< 0.001
log(n Votes for Democratic Presidential Candidate)	0.0046	0.0017	0.006
log(n Votes for Republican Presidential Candidate)	-0.0043	0.0022	0.050
Ideological Distance	0.1039	0.0278	< 0.001
Democrat Candidate's CF Score	0.0150	0.0046	0.001
Republican Candidate's CF Score	0.0116	0.0055	0.033
Democratic Incumbent	0.0052	0.0061	0.396
log(Voting Eligible Population)	0.0544	0.0060	< 0.001
Year = 1986	-0.0022	0.0019	0.256
Year = 1988	0.0158	0.0067	0.019
Year = 1990	0.0060	0.0062	0.336
Year = 1992	0.0111	0.0072	0.126
Year = 1994	0.0094	0.0062	0.129
Year = 1996	-0.0222	0.0064	< 0.001
Year = 1998	-0.0044	0.0072	0.538
Year = 2000	-0.0035	0.0066	0.599
Year = 2002	0.0007	0.0067	0.922
Year = 2004	-0.0037	0.0067	0.584
Year = 2006	0.0058	0.0064	0.370
Year = 2008	0.0307	0.0072	< 0.001
Year = 2010	0.0120	0.0071	0.094
Year = 2012	-0.0303	0.0076	< 0.001
Year = 2014	0.0023	0.0079	0.773
Year = 2016	-0.0235	0.0069	< 0.001
Year = 2018	-0.0029	0.0084	0.730
Bottom 5%	-0.0003	0.0079	0.968
Top 5%	-0.0230	0.0078	0.003
Middle 90%	0.0005	0.0001	< 0.001
Bottom 5% $\times$ Dem. Spending Adv.	0.0213	0.0075	0.005
Top 5% $\times$ Dem. Spending Adv.	0.0004	0.0001	< 0.001
Lagged Outcome	0.1785	0.0160	< 0.001

$n = 481$.

Appendix G: KRLS with Dichotomous Outcomes

In this section, we replicate our analyses now replacing our outcome variable with an indicator for whether the Democratic party candidate won a seat. These average marginal effects are therefore not strictly comparable, since the outcome variable has changed from the vote share (between 0 and 1, averaging about 0.5) to a dichotomous 0–1 value. Results here are therefore interpretable as marginal effects on the probability of a Democrat winning a seat. Again, our results persist. In terms of probability, the average marginal effect of *Democratic Expenditure Advantage* is about 11% in the House and about 2.4% in the Senate. Both remain highly statistically significant.

Table S9: House KRLS Average Marginal Effects, Dichot. Outcome

Variable	Estimate	SE	p
Democratic Spending Advantage	0.1070	0.0045	< 0.001
log(Total Spending)	0.0062	0.0085	0.471
Democratic Presidential Vote Advantage	0.0066	0.0003	< 0.001
Ideological Distance	0.3634	0.0507	< 0.001
Democrat Candidate's CF Score	0.0219	0.0064	< 0.001
Republican Candidate's CF Score	0.0318	0.0076	< 0.001
Dem. Inc./Low Qual. Chall.	0.2289	0.0069	< 0.001
Dem. Inc./High Qual. Chall.	0.2029	0.0104	< 0.001
Rep Inc./Low Qual. Chall.	−0.2164	0.0068	< 0.001
Rep Inc./High Qual. Chall.	−0.1863	0.0100	< 0.001
Open Seat/Both High Qual.	−0.0267	0.0147	0.069
Open Seat/High Qual. Dem/Low Qual. Rep	0.0954	0.0161	< 0.001
Open Seat/Low Qual. Dem/High Qual. Rep	−0.1756	0.0162	< 0.001
Year = 1980	−0.0373	0.0114	0.001
Year = 1982	0.0553	0.0107	< 0.001
Year = 1984	−0.0142	0.0111	0.201
Year = 1986	0.0197	0.0109	0.070
Year = 1988	0.0048	0.0107	0.653
Year = 1990	0.0261	0.0112	0.020
Year = 1992	0.0088	0.0101	0.381
Year = 1994	−0.0578	0.0101	< 0.001
Year = 1996	0.0201	0.0096	0.037
Year = 1998	0.0093	0.0114	0.417
Year = 2000	−0.0069	0.0107	0.522
Year = 2002	−0.0031	0.0114	0.788
Year = 2004	−0.0031	0.0105	0.766
Year = 2006	0.0292	0.0107	0.006
Year = 2008	0.0206	0.0109	0.059
Year = 2010	−0.0511	0.0105	< 0.001
Year = 2012	0.0063	0.0110	0.567
Year = 2014	−0.0225	0.0114	0.049
Year = 2016	−0.0116	0.0126	0.356
Year = 2018	0.0162	0.0123	0.188
Bottom 5%	−0.1058	0.0105	< 0.001
Middle 90%	0.0026	0.0068	0.700
Top 5%	0.1011	0.0107	< 0.001
Bottom 5% × Dem. Spending Adv.	0.0144	0.0010	< 0.001
Top 5% × Dem. Spending Adv.	0.0140	0.0010	< 0.001

$n = 5859$.

Table S10: Senate KRLS Average Marginal Effects, Dichot. Outcome

Variable	Estimate	SE	p
Democratic Spending Advantage	0.0236	0.0022	< 0.001
log(Total Spending)	0.0423	0.0244	0.084
Democratic Presidential Vote Advantage	0.0047	0.0006	< 0.001
Adj. Dem. Pres. Vote Advantage	0.0077	0.0008	< 0.001
log(n Votes for Democratic Presidential Candidate)	0.0173	0.0079	0.029
log(n Votes for Republican Presidential Candidate)	−0.0145	0.0105	0.170
Ideological Distance	0.5463	0.1363	< 0.001
Democrat Candidate's CF Score	0.0033	0.0215	0.878
Republican Candidate's CF Score	0.0805	0.0257	0.002
Open Seat	0.0143	0.0306	0.640
Democratic Incumbent	0.2890	0.0274	< 0.001
log(Voting Eligible Population)	−0.0086	0.0086	0.315
Year = 1980	−0.1076	0.0350	0.002
Year = 1982	0.0222	0.0358	0.537
Year = 1984	0.0386	0.0367	0.293
Year = 1986	0.0846	0.0365	0.021
Year = 1988	0.0240	0.0335	0.474
Year = 1990	0.0138	0.0391	0.725
Year = 1992	0.0356	0.0336	0.290
Year = 1994	−0.0633	0.0350	0.071
Year = 1996	−0.0299	0.0364	0.412
Year = 1998	0.0068	0.0360	0.849
Year = 2000	0.0293	0.0363	0.419
Year = 2002	−0.0216	0.0370	0.559
Year = 2004	−0.0288	0.0354	0.417
Year = 2006	0.1058	0.0385	0.006
Year = 2008	0.0639	0.0379	0.093
Year = 2010	−0.0982	0.0401	0.015
Year = 2012	0.0483	0.0407	0.236
Year = 2014	−0.0846	0.0371	0.023
Year = 2016	−0.0375	0.0436	0.390
Year = 2018	0.0216	0.0422	0.609
Bottom 5%	−0.1169	0.0275	< 0.001
Middle 90%	0.0130	0.0178	0.465
Top 5%	0.0912	0.0259	< 0.001
Bottom 5% × Dem. Spending Adv.	0.0014	0.0004	< 0.001
Top 5% × Dem. Spending Adv.	0.0014	0.0004	< 0.001

$n = 586$.

Appendix H: Replication w/ SVM & Nonparametric Bootstrap

In this section, we replicate all our analyses using support vector machine regression. This replication analysis serves as a robustness check for two reasons. First, we employ a different machine learning technique to estimate marginal effects and hypotheticals. Second, we employ an entirely different statistical inference technique. Whereas we relied on the parametric bootstrap for our KRLS results, SVM does not produce an estimate of the variance-covariance matrix.

Therefore, we used the nonparametric bootstrap. First, we resample from each dataset, training an SVM model. Second, we predict outcomes from our original datasets for each hypothetical spending schedule, including observed spending levels. We repeat these two steps 1000 times. Finally, we use the resulting bootstrap distribution to perform analogue analyses to those presented in the main paper.

Appendix Figure S3 replicates Figure S1, now using the nonparametric bootstrap. The confidence intervals depicted in this figure are subtly different from those in the main paper and in Figure S1. In the main paper, we use the standard LOESS confidence intervals, which rely on the normal approximation. Here, we identify the 95% bootstrap confidence interval, fitting a LOESS smoothed line for each resample, using that model to get fitted values for our full dataset, and then using the resulting 95% confidence intervals for inference. Neither the method used here, nor the one in the paper, should be regarded as more correct, and their difference helps consolidate the case for our main inference: the marginal effect of spending is nonlinear, exactly as would be expected according to the theory of contest success functions. Moreover, in the paper, we reported the approximate integrals under each of these curves; for KRLS, they were 9% for the House and 10% for the Senate. Using SVM, these figures are larger: 14% for the House and 10% for the Senate.

Appendix Figure S4 replicates Figure 2 from the paper, which compares two hypothetical spending profiles, one (darker/blue) in which all races have *Democratic Expenditure Advantage* held at its 95th percentile, and another (lighter/red) in which all races have *Democratic Expenditure Advantage* held at its 5th percentile. The results are broadly similar to those in the paper. The similarity is sharper for the Senate than for the House, but in both methods warrant the same inference: these spending levels are sufficient to purchase control of Congress.

Appendix Figure S5 replicates Figure 5 from the paper, which compares two hypothetical spending profiles, one at actual spending levels and another that zeroes out spending advantages and holds *Total Expenditures* at its minimum observed levels. Again, the results are broadly similar to those in the paper. The similarity remains sharper for the Senate than for the House.

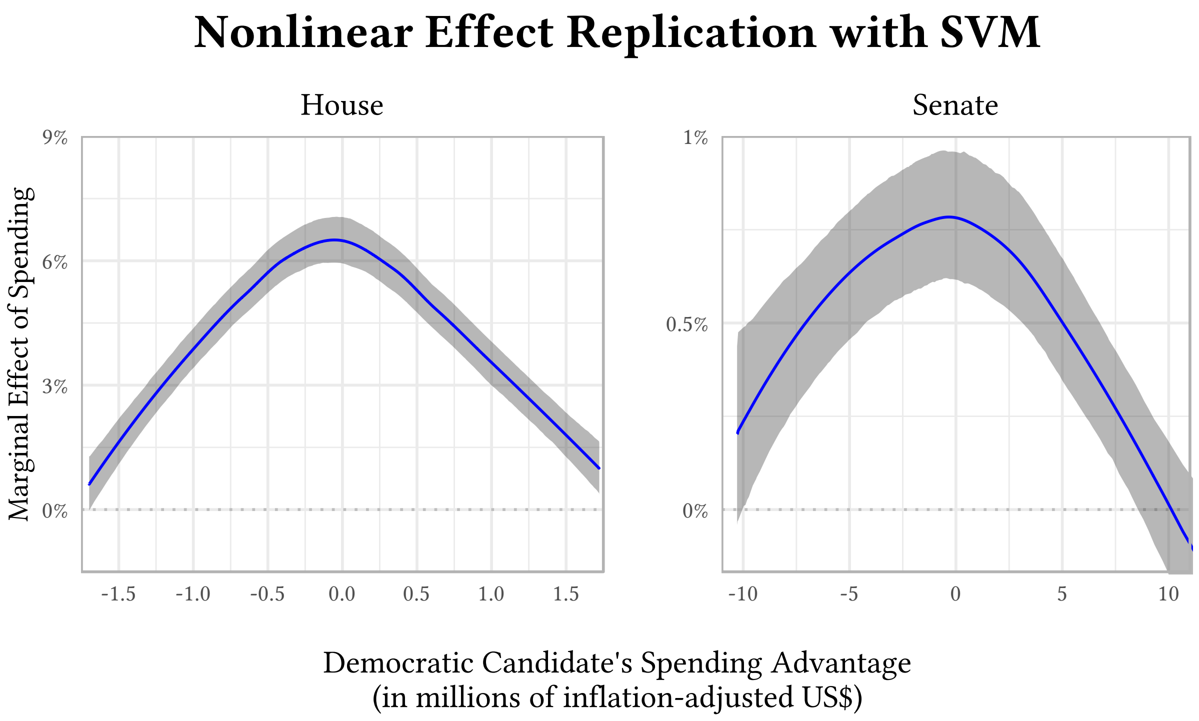

Figure S3: Replication of Figure S1 with SVM and the nonparametric bootstrap.

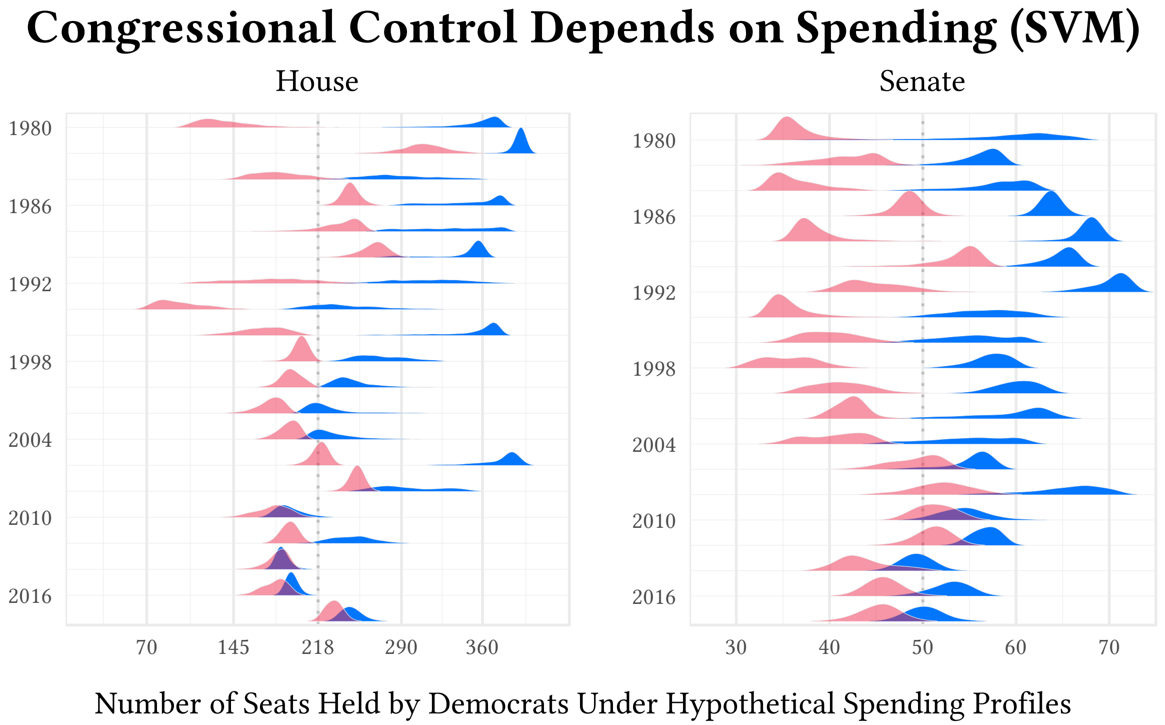

Figure S4: Replication of Figure 2 from the main paper with SVM and the nonparametric bootstrap. Densities indicate simulated distributions of the numbers of seats held by Democrats under the hypothetical cases with Democrats' advantage held at the 95th percentile (darker/blue) and with Democrats' advantage held at the 5th percentile (lighter/red).

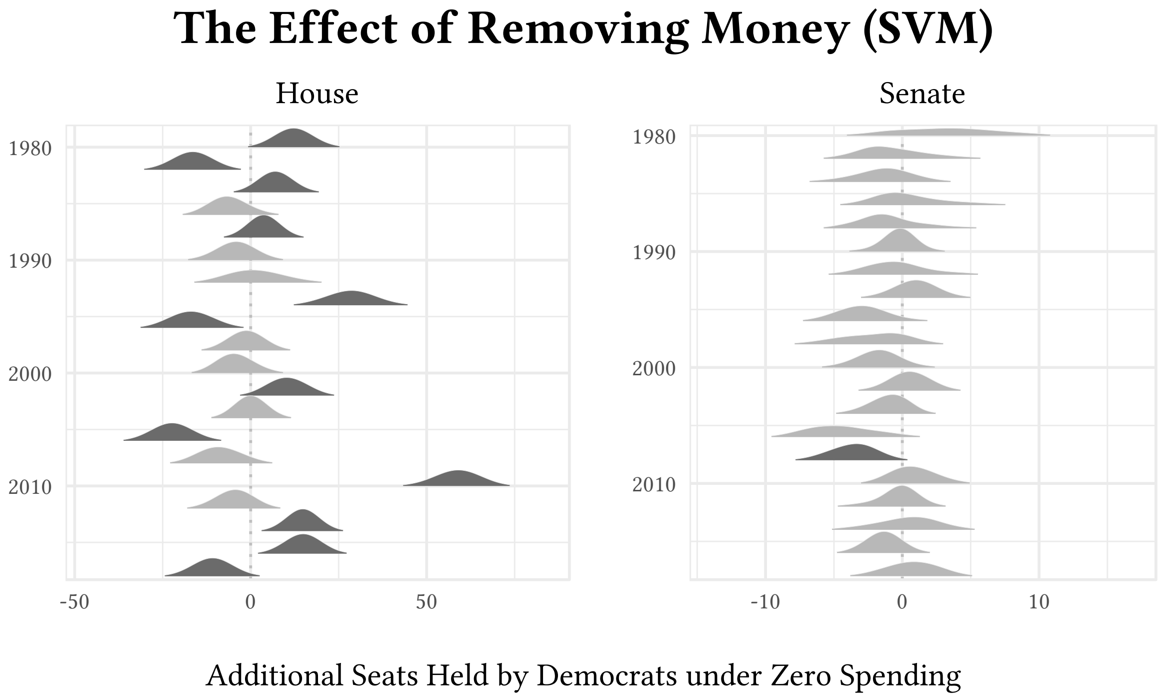

Figure S5: Replication of Figure 5 from the main paper with SVM and the nonparametric bootstrap. Densities indicate simulated distributions of the numbers of seats held by Democrats under the hypothetical zero spending case minus that under the actually observed case. Dark gray densities indicate year-chambers in which the 95% interval excludes zero.

References

1. Abramowitz, A. Explaining Senate Election Outcomes. American Political Science Review. 1988;82(2):385–403.
2. Ensley, M. Individual campaign contributions and candidate ideology. Public Choice. 2009;138:221–238.
3. Mohanty P, Shaffer R. Messy Data, Robust Inference? Navigating Obstacles to Inference with bigKRLS. Political Analysis. 2019;45(2):127–144.
